# The Quality of Life among Men Receiving Active Surveillance for Prostate Cancer: An Integrative Review

**DOI:** 10.3390/healthcare7010014

**Published:** 2019-01-22

**Authors:** Sabrina L. Dickey, Ciara J. Grayson

**Affiliations:** 1 College of Nursing, Florida State University, Tallahassee, FL 32306, USA; 2 College of Medicine, Florida State University, Tallahassee, FL 32306, USA; cjg12b@med.fsu.edu

**Keywords:** active surveillance, prostate cancer, quality of life, health related quality of life

## Abstract

Prostate cancer is very common among men in the United States. The current literature on active surveillance (AS) suggests that it is a promising treatment option for men with low-risk prostate cancer. The purpose of this manuscript is to provide a thorough integrative review regarding the effects of AS on the quality of life (QoL) of men with prostate cancer. Utilizing a methodological strategy, electronic databases were reviewed for empirical articles during the time frame of January 2006 to December 2016. A total of 37 articles met the inclusion criteria wherein 20 focused on the QoL among men only receiving AS and 16 reported QoL among men undergoing AS and other forms of treatment for prostate cancer. The review highlights the purpose, common instruments, race and ethnicity, and strengths and limitations of each article. The majority of articles indicated low levels of anxiety and depression and decreased incidences of bladder, bowel and sexual functioning among men undergoing AS in comparison to men who received other treatment modalities. The results indicated that additional research is needed to determine the QoL among men receiving AS on a longitudinal basis. The results support previous literature that indicated the positive impact of AS on low-risk prostate cancer.

## 1. Introduction

Prostate cancer is the most common non-skin cancer found in American men [1,2]. Over the past three decades, prostate cancer diagnoses have risen dramatically [3]. The majority of men with prostate cancer remain clinically asymptomatic throughout their life [4]. Even though the data show that men diagnosed with prostate cancer typically die from reasons unassociated with their disease, most patients still opt for aggressive treatment [5]. Some of the common treatment options available for prostate cancer are brachytherapy, radiation, and radical prostatectomy. However, the common treatment options for prostate cancer bear specific risks that affect an individual′s quality of life (QoL) and/or health related quality of life (HRQoL) [6,7]. The invasive nature of some treatments leads to negative impacts on sexual, bowel, and urinary function, which in turn causes difficulties between those diagnosed with prostate cancer and their partners [6,7]. The one treatment option for prostate cancer that does not require chemotherapy, radiation, or surgery is active surveillance (AS) along with watchful waiting. At one time AS was underutilized, however, recent research indicates that it is increasingly used for prostate cancer in the United States [8,9,10,11]. Despite the benefits of AS as a treatment for prostate cancer, it remains an underutilized treatment modality in the United States (USA) [8].

### 1.1. Prostate Cancer

Prostate cancer is the sixth leading cause of cancer death worldwide [12]. In the USA, the estimated incidence of prostate cancer in 2016 was 180,890, which accounted for 21% of cancer diagnoses with regards to men [13]. The aggressive treatment of otherwise indolent prostate cancer tumors exposes individuals to potentially life altering side effects from the treatment [14]. Consequently, the potential for significant side effects from prostate cancer treatments led physicians to recommend AS as a treatment option [15].

In 2017, the American Cancer Society indicated that the 5-year relative survival rate for prostate cancer is 99%, which indicated that AS can be a promising treatment for nonaggressive forms of prostate cancer [2]. It is important to note that prostate cancer will progress. However, some tumors will progress at slower rates [3]. Therefore, in most cases, the tumor itself does not pose a threat to patients [5]. 

### 1.2. Active Surveillance

According to the American Cancer Society, AS is when the cancer is monitored carefully by a physician, which includes a prostate specific antigen test and a digital rectal exam every six months and prostate biopsies every year [2]. Active surveillance has been used interchangeably with the term “watchful waiting”. However, the literature indicated a separate distinction between the two terms. Watchful waiting, as a treatment for prostate cancer, is indicated for those with advanced prostate cancer, a limited life expectancy, and the goal of being palliative and not curative [8,16,17]. In contrast, AS, is individualized with a longer life expectancy and the goal of being curative if the cancer progresses [8,14,16,17,18]. 

During AS, the health care provider follows a protocol that does not involve surgery, chemotherapy, hormone treatment, or radiation to monitor the growth of the prostate cancer. The AS protocols vary among health care providers as there is no established protocol that is accepted by all health care providers. For example, research in Toronto Canada indicated an AS protocol which consisted of PSA testing every 3 months for 2 years, then every 6 months with a biopsy during the first year followed by every 3 to 4 years until age 80 [19]. The John′s Hopkins AS protocol included PSA testing and DRE every six months and a yearly prostate biopsy [19]. Despite the lack of treatments when AS is used, if signs of significant disease progression occur, the patient can undergo radical treatment at any time [20]. During AS, treatment is deferred until the tumor becomes clinically significant, which oftentimes never happens [3]. According to Surveillance, Epidemiology, and End Results and Medicare data, the use of AS rose from 9.7% in 2004 to 15.3% in 2007 [6]. In a study that examined the trends of prostate cancer treatment via Cancer of the Prostate Strategic Urologic Research Endeavor (CaPSURE) database, it was reported that AS use for low-risk disease increased from 6.7–14.3% from 1990–2009 to 40.4% from 2010–2013 [21]. Similarly, according to the American Urological Association Quality (AQUA) Registry, which included 47,288 prostate cancer patients from 2014–2016, AS rates for low-risk disease rose [22]. 

Research continues to examine whether AS is a safe treatment option with benefits for men with low-risk prostate cancer. Active surveillance was deemed as a safe alternative to radical treatment in a study that inquired whether anxiety and depression developed among men treated with AS2 [23]. Haymart and colleagues (2017) reported that the 5-year survival rate of nearly 100% for prostate cancer, along with the possible reduction in treatment side effects, indicated AS as a promising treatment for prostate cancer [24]. Similarly, in a comparison of radical prostatectomy, radiotherapy, and AS for prostate cancer, AS proved more beneficial for better sexual and urinary function in comparison to curative treatments [23]. Consequently, AS is a promising treatment option for those with low-risk prostate cancer which eliminates the need for radical treatments, which can cause anxiety and a decrease in QoL [25]. In the Prostate Cancer Intervention versus Observation Trial (PIVOT) study that followed patients for up to 20 years, radical prostatectomy all-cause mortality and prostate-cancer specific mortality were not significantly lower than observation in patients with localized prostate cancer (*p* = 0.06) [26]. It can be concluded that AS is a safe treatment option and that the risk of progression to metastatic disease and mortality is low. However, it is important to note that this depends on the low-risk classification protocol, which may vary slightly by institution. To date, the best treatment option for men with low-risk prostate cancer remains unclear [26]. Some of the ambiguity may arise from institutional differences in which patients meet the requirements for AS as a treatment option. The uncertainties regarding which treatment option is best strengthens the importance of this review, which further explores AS and its effects on QoL. 

#### Active Surveillance among African Americans

It is important to note that while AS is a promising treatment option for patients with low-risk prostate cancer, African American men are affected at higher rates and have more aggressive forms of prostate cancer [27]. The mortality risk in African American men is reportedly 2.4 times that of White men [27]. Additionally, African Americans experience higher disease progression than their White counterparts [28]. This health disparity reduces the benefit of AS for African Americans living with prostate cancer because they are more likely to experience disease progression. While many studies have shown that AS has a decreased negative impact on QoL [6,29,30,31,32], most of them include less than 10% of African American men in their subject population [33]. Due to the disproportionately low numbers of African Americans in the studies, the results cannot be generalized to African American men [27]. Additionally, African Americans who chose to undergo AS were more likely to experience disease progression compared to White men [33]. It has been suggested that African Americans and other minority populations should have a more stringent criteria to qualify for AS [33].

### 1.3. Quality of Life

The mere diagnosis of cancer can significantly change the life of a patient and their family [34]. The thought of living with untreated cancer can possibly cause psychological distress and anxiety [35]. However, few studies have found significant negative psychological impacts for patients undergoing AS [34]. Quality of life and HRQoL are often used when comparing prostate cancer treatments [6]. Quality of life seemed to be the broad term that encompassed the physical and psychological aspects associated with treatment for prostate cancer, such as, urinary function and bother, sexual function and bother, bowel function and bother, and hormone function and bother [6,20,29]. The terms QoL and HRQoL are similar in that they are often used to gauge the psychosocial and physical outcomes of prostate cancer survivors. Follow up care for cancer survivors not only includes the treatment outcome, but also an overall improvement in their health in relation to their QoL [36]. For the purposes of the current review, QoL will be utilized as the overarching term for the physical and psychosocial outcomes associated with QoL and HRQoL prostate cancer survivors.

The concept of QoL is an essential aspect in the lives of prostate cancer survivors. Previous reviews regarding AS among prostate cancer survivors compared AS vs surgery and/or radiation, the psychosocial impact of AS on survivors and couples, and the under-utilization of AS [8,37,38,39]. However, there were a lack of integrative reviews, which is a methodology that allows for the inclusion of various types of studies as opposed to primarily only randomized clinical trials. Additional studies, which are long term in nature and consist of diverse patient samples, are needed for the continued examination of the impact of AS on the prostate cancer survivor′s QoL. The purpose of this paper is to provide an integrative review of the literature regarding the effects of AS as a treatment option for a prostate cancer survivor′s QoL. 

## 2. Methods

### 2.1. Integrative Review Methodology

This study employed Whittemore and Knafl′s (2005) methodological strategies for conducting an integrative review [40]. The purpose of this integrative review was to synthesize the occurrence of literature regarding QoL among men receiving AS for prostate cancer between 2006 and 2016. Therefore, a quality appraisal was not implemented. The selected methodology of this review entailed a process of problem identification, literature search, data evaluation, data analysis, and presentation of the literature. This approach examined empirical, theoretical literature, and non-experimental studies related to the concept being researched. The inclusion of various types of literature, such as, theories, concepts, and pertinent issues within the healthcare arena is a benefit of integrative reviews. In addition, the method combined comprehensive searching with purposeful sampling to ensure all relevant literature was identified [40]. 

### 2.2. Search of the Databases

The electronic databases of Cinhal, Google Scholar, ProQuest, and PubMed were selected to examine the literature. The EMBASE database was not utilized in the review due to the University′s lack of subscription to the database. Each search of the databases utilized the key words “active surveillance”, “prostate cancer”, “cancer”, “quality of life”, and “health related quality of life”. The keywords were entered into each of the databases in different combinations that were established by the authors. The authors followed an identical format in searching the databases to ensure consistency with the data search. A search of the databases took place during January and April 2017 to capture relevant articles for the review. The inclusion criteria for this review were empirical or theoretical articles published between 2006 and 2016, written in the English language, and focused solely on the QoL factors of men undergoing AS as a treatment for prostate cancer. As indicated in the data abstraction process by Whittemore and Knafl (2005), the reference lists were also reviewed for inclusion. The study design was not a limitation of the review, which included quantitative, qualitative, mixed methods studies, and systematic reviews [40]. The exclusion criteria consisted of articles that were non-English, commentaries, dissertations, theses, editorials, letters to the editor, and books. Articles published outside of the time frame of 2006–2016 were excluded. 

### 2.3. Data Analysis

**Figure 1:**
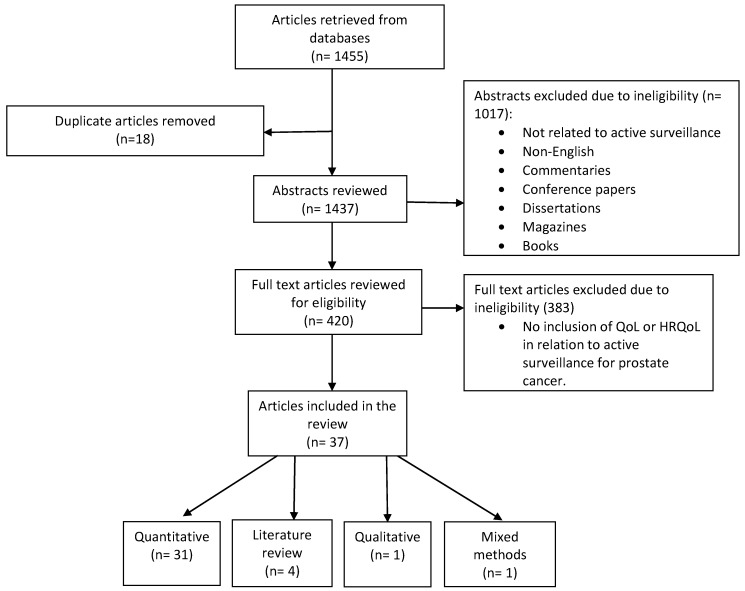
Data flow chart of the article selection process.

The two authors independently evaluated the databases for articles that met the inclusion criteria. Each of the authors developed a table of their results and compared their findings. The results were used to determine which of the articles met the inclusion criteria and warranted further analysis to meet the objective of the review. Figure 1 represents a flow chart of the article selection process. 

## 3. Results

The current integrative review contained 37 peer reviewed empirical articles that examined the QoL among men undergoing AS for prostate cancer. A preliminary review of the abstracts from the selected electronic databases yielded 1455 articles. The authors compared and discussed their findings in each step of the data analysis in order to provide a thorough analysis. After the removal of duplicates (*n* = 18), the abstracts were screened (*n* = 1437) for inclusion in the review. Pertinent articles included studies that measured the physiological and psychological aspects of QoL among men receiving AS for prostate cancer. Upon review of the abstracts, 1017 were excluded (commentaries, books, non-English, etc.). Next, a review of the full text was implemented on the remaining articles (*n* = 420), in which four were identified from references and 383 did not meet the inclusion criteria (lack of AS in relation to QoL). 

A thorough review of the remaining articles yielded a final count of 37 that met the inclusion criteria for the current review. An overview of the articles indicated that the majority were quantitative studies, i.e., a total of 31. Literature reviews were the next largest category (*n* = 4), followed by qualitative (*n* = 1), and mixed method (*n* = 1) articles. The selected articles were summarized in tables according to their theme (physiological and psychological). Each table included the author′s name, purpose, setting, design, race/ethnicity, methods, statistics, sample, response rate, reported instruments, key findings, strengths, and limitations. To minimize bias, two reviewers independently developed separate electronic spreadsheets of the articles that met the inclusion criteria for the integrative review. The reviewers discussed and compared their findings until a consensus was reached regarding the inclusion of the articles in the integrative review. 

All the quantitative studies utilized validated surveys and questionnaires to gather information regarding QoL. The most commonly used questionnaires that measured QoL in the articles consisted of eight that measured QoL among prostate cancer survivors, the Expanded Prostate Cancer Index Composite (EPIC), five studies that used the Functional Assessment of Cancer Therapy-Prostate (FACT-P), and four that contained the European Organization for Research and Treatment of Cancer (EORTC) to name a few. Sexual function was a pertinent theme in the articles wherein six measured erectile dysfunctions with the International Index of Erectile Function (IIEF). Lastly, anxiety and depression were factors that influenced QoL among prostate cancer survivors receiving AS and six articles utilized the Hospital Anxiety and Depression Scale (HADS) to measure anxiety and depression. Overall, the questionnaires assessed various issues among the prostate cancer survivors such as anxiety, depression, physical strength, and their overall well-being as prostate cancer survivors. 

A synopsis of the 37 articles reviewed indicated that 26 focused on the combination of the QoL factors of sexual, bladder, and bowel function, and anxiety and depression. The remaining 11 articles encompassed the QoL factors of anxiety and depression. Therefore, the data from the review was divided into two themes regarding QoL among AS articles: (1) combined physiological and psychological factors (sexual, bladder, bowel function, and anxiety and depression, and (2) only psychological factors (anxiety and depression). 

The majority (86%; *n* = 32) of the articles in the review were non-experimental/cross-sectional, wherein there was only one randomized clinical trial (Lane, 2016). The sample size in the studies ranged from seven to 1643. The racial distribution of the studies consisted of mostly white men and revealed a significant lack of minority populations. For example, the racial distribution for the study conducted by Parker et al. (2016) was 86.1% White, 6.7% Black, 6.1% Hispanic, and 1.1% Asian [16]. Similar ratios regarding white and minority participants were found in other studies with less than 10% identified as minorities [41,42] or of a race that was not addressed in the studies [43,44,45,46,47,48]. 

The next sections of the integrative review will discuss the common QoL themes identified in the articles. Data from the two themes have been synthesized into two separate tables. The theme of combined physiological and psychological factors (sexual function, and bladder and bowel function, and anxiety and depression will be discussed first.

### 3.1. Sexual, Bladder, and Bowel Function and Anxiety and Depression Factors of QoL among Active Surveillance and Other Forms of Treatments 

The current theme was comprised of articles that examined QoL through the factors of anxiety and depression, sexual function, and bladder and bowel functions of men who received AS and other forms of treatments for prostate cancer. A total of 26 articles were identified for the theme, which was comprised of 18 regarding sexual, bladder, and bowel function, six focused on a combination of anxiety, depression, sexual, bladder, and bowel function, one regarding only bladder and bowel function, and one focused on sexual function. Various studies examined the impact of non-AS treatment options on the prostate cancer survivors′ factors of sexual, bladder, and bowel function [6,29,31,32,45,46,49,50,51]. Common non-AS treatment options indicated in the articles consisted of, focal cryoablation, brachytherapy, external beam radiotherapy, and radical prostatectomy, which evaluated the effects of QoL on prostate cancer survivors [6,29,32,49,50,52]. The intimate topics of erectile dysfunction and sexual functioning were topics that surfaced and impacted the QoL of prostate cancer survivors. 

#### 3.1.1. Erectile and Sexual Function Factors of QoL among Active Surveillance and Other Forms of Treatments

When evaluating the sexual function of prostate cancer survivors, it would be remiss not to include erectile function. Erectile functioning emerged as a pertinent aspect of sexual functioning within the articles. For example, a study comparing focal cryoablation, brachytherapy, and AS reported men undergoing AS had lower mean scores on an erectile function questionnaire [46]. Donovan et al. (2015) compared AS to radical prostatectomy and radical radiotherapy and reported that erectile function decreased in all groups [31]. However, it decreased considerably more in the radical radiotherapy and radical prostatectomy groups. Similar results were found among a study that compared watchful waiting to brachytherapy and radiotherapy, primary hormonal therapy, and prostatectomy [32]. The study was comprised of participants that engaged in AS and watchful waiting. However, the researchers could not distinguish which of the treatments were used by the participants when treatment was deferred. Therefore, the researchers classified all the participants, whether they received AS or watchful waiting, as receiving watchful waiting. The results indicated that patients undergoing watchful waiting were less likely to report impotence, which was an indicator for measuring QoL [32]. 

In contrast, Pham et al. (2014) reported statistically significant (*p* < 0.05 at 1 year and 2 years) lower levels of erectile function among men receiving AS when compared to a control group which consisted of patients without prostate cancer [51]. Similarly, a study regarding the impact of prostate cancer biopsies among men receiving AS for prostate cancer indicated a small decrease in sexual function over time [53]. An additional study in the review that examined QoL among men only receiving AS also found a small decrease in sexual function which persisted every 6 months for 30 months [16]. A decrease in erectile and sexual function among prostate cancer survivors receiving AS was in the minority in the articles in the review. 

The loss of sexual functioning has physiological and psychological implications on the QoL in the lives of men, as well as their partners. The decreased invasiveness of AS in the treatment of low-risk prostate cancer is a factor which promotes men maintaining erectile function. A comparison of AS to radical prostatectomy indicated men on AS had significantly better sexual functioning [6]. In a study that compared AS to brachytherapy and laparoscopic prostatectomy, sexual function decreased in 30% of patients on AS, 59% of patients who underwent brachytherapy, and 71% of patients who underwent laparoscopic prostatectomy [29]. Likewise, additional articles that examined QoL among men with localized prostate cancer reported sexual functioning as significantly better (*p* < 0.01) and there were higher scores for normal sexual functioning among those undergoing AS when compared to radical prostatectomy or radiation therapy [45,50]. However, the studies indicated that over an extended period, treatment modalities other than AS can offer the same degree of QoL among the issue of sexual functioning. Punnen et al. (2013) conducted a longitudinal study that compared AS to various treatment modalities, (radical prostatectomy, brachytherapy, external beam radiotherapy, and primary androgen deprivation therapy) to examine long term QoL [41]. The results suggested that QoL equalized over time. For example, despite an initial decreased QoL among the majority of the men in the study and decreased sexual function over the first two years among all forms of treatment, there was minimal change from three to ten years [41]. 

#### 3.1.2. Bladder and Bowel Factors of QoL among Active Surveillance and Other Forms of Treatment

Bladder and bowel scores in relation to QoL were prevalent among many of the articles in the review that contained different modalities of treatment for prostate cancer. Concern regarding urinary functioning was a prevalent issue in the lives of prostate cancer survivors. Urinary function outweighed issues pertaining to sexual function for a cohort of men undergoing AS in Ireland [54]. In fact, uncertainty among urinary function showed no improvement after a five-week follow-up that occurred after an internet intervention to reduce uncertainty for men on AS [55]. In the study by Lokman et al. (2015), there was no decrease in the QoL regarding urinary function among men who received AS [56]. Similar results were found in one of the articles that reported no changes in urinary function or QoL among Finnish men receiving AS for prostate cancer [57]. A higher baseline score for urinary function among men receiving AS was reported in a study that compared QoL for men receiving AS and men who had negative biopsy results for prostate cancer [58]. However, using a model which estimated changes over time indicated a decrease in urinary function among the men who received AS [58]. A comparison of QoL among men receiving AS in Ireland and the USA revealed differences in urinary scores between the two populations [54]. The men from Ireland reported a higher mean (84.4) for urinary bother compared to (71.4) among men in the USA [54]. 

Along with concern regarding urinary function among prostate cancer survivors was the issue of bowel function within some of the articles in the review. Bowel and bladder function seemed to be inextricably linked as a factor for assessing QoL among men with prostate cancer. In a study that examined QoL among men receiving AS and radiation therapy, there were declines in QoL and worse bowel and urinary bother functions that yielded significant results, (*p* < 0.05) among men who received radiation therapy [59]. Quality of life regarding bowel and bladder function appeared to be maintained in a study among men who were treated with surgery, active monitoring, and radiotherapy [31]. Nevertheless, there were initial decreases in the QoL due to bladder and bowel issues during the first months of treatment that utilized surgery and radiotherapy [31]. In a comparison of QoL among men receiving AS for prostate cancer and men without prostate cancer, the results indicated a decline in bowel and urinary function for the men on AS [58]. However, the decline in men receiving AS was a comparison to men without cancer, which provided some insight into why bowel function scores may have been lower [58]. A five-year follow-up study among men undergoing AS revealed no changes among bowel function and other factors for QoL [60]. The study indicated that treatment may be linked to changes in bowel, urinary, and sexual function [60].

#### 3.1.3. Combination of Physiological and Psychological Factors of QoL among Active Surveillance and Other Forms of Treatment

Lokman et al. (2013) examined the impact of AS on the QoL factors of erectile and urinary function among low-risk prostate cancer patients [56]. The results indicated there was no decrease in erectile or urinary function among the men after one or three years of follow-up [56]. A similar study also examined AS and QoL based on erectile and urinary function among low-risk prostate cancer survivors [57]. The study reported no significant changes in erectile and urinary function among prostate cancer survivors one year after following up. An additional article in the review found no significant difference in the QoL factors of sexual or urinary function among prostate cancer survivors, prior to and at the onset of receiving AS [61]. The findings from this review suggest that AS is positively related to the QoL factors of erectile and urinary function.

The combination of anxiety and decreased sexual functions were prevalent in the articles reviewed in the current review [16,57,58,60,61,62]. A study was conducted to examine the mental and physical QoL factors among Finnish men receiving AS for low-risk prostate cancer [57]. The QoL was not affected and anxiety did not cause the men to change their choice of treatment. In fact, the results were compared to the general population based on age, which indicated better overall mental and physical health among the men who received AS [57]. A first of its kind study in Australia examined QoL and anxiety among a cohort of men receiving AS for prostate cancer [61]. The QoL scores did not decrease and the men reported low levels of anxiety. Similar results were found in the review among the study by Seiler et al. (2012), which reported that men receiving AS exhibited low levels of anxiety and prostate cancer anxiety that were below the clinical levels. Additionally, the partners of the men receiving AS also experienced low distress and low levels of anxiety that were not significant [62]. Based on the results, one can surmise the prostate cancer patient′s QoL is impacted by the combination or separate factors of sexual function and anxiety. 

Disease uncertainty, anxiety, and the fear of progress were also examined for a relationship among QoL and prostate cancer patients who received AS for prostate cancer. In the study, an inverse relationship existed among PSA scores and urinary and bowel QoL scores. Increased PSA scores were associated with decreased urinary and bowel scores. Similarly, as uncertainty scores increased, the QoL factors of urinary, bowel, sexual, hormonal, and satisfaction decreased. Lastly, increased anxiety predicted lower urinary, bowel, sexual, hormonal, and satisfaction QoL scores [16]. 

One of the recent largest studies in the United Kingdom examined patient reported outcomes (PROMS) from a randomized trial, Prostate Testing for Cancer Treatment (PROTEC T). The PROTEC T trial compared the use of AS, radical prostatectomy, and external beam radiotherapy with other segments of the population for the treatment of localized prostate cancer. Active surveillance was tied with radiotherapy as being the second largest category for treatment of prostate cancer, (*n* = 545), [30]. Obtaining the PROMS from patients with prostate cancer is essential for selecting the appropriate form of treatment and managing the impact of the treatment on their QoL [30]. Additionally, the PROMS provided an insight into the progression of the disease and the patient′s mortality from the disease [30]. The results from the PROTEC T indicated men on AS had the lowest scores for issues with sexual function (22.8%), lowest score for small overall sexuality problems (23%) and tied with all categories for big/moderate erectile problems (16%) [30]. However, results for anxiety, depression, health utility, mental, and physical health, were higher among the men receiving AS. The increased rates of anxiety and depression among receiving AS was also found in one of the articles included in the current review [42]. These findings indicated the negative impact that living with untreated prostate cancer can have on the QoL of men. 

The results indicate the subjective nature of QoL among various populations of men along with the understanding that there is often a combination of factors, such as sexual, bladder, and bowel function and anxiety upon examining QoL among men with prostate cancer. Longitudinal research that assesses QoL among prostate cancer patients receiving AS will provide additional support for the benefits of receiving AS. The results of this section are presented in Table 1.

### 3.2. Anxiety and Depression Factors for QoL among Active Surveillance Treatment

The factors of anxiety and depression amongst patients receiving AS was a common theme throughout many of the articles. The psychological impact of living with prostate cancer has the potential to lead towards the development of anxiety and depression. There was a total of 11 studies that examined anxiety, prostate cancer specific anxiety, and depression among men who utilized AS. Additionally, all the studies were quantitative in methodology. 

The most commonly used instruments to measure anxiety amongst the articles were the State-Trait Anxiety Inventory scale (STAI) and the Memorial Anxiety Scale for Prostate Cancer (MAX-PC). The STAI and MAX-PC were used in conjunction or separately in five studies. Depression was assessed most frequently with the Hospital and Anxiety Depression Scale (HADS) and/or Centre for Epidemiologic Studies Depression Scale (CES-D). Additionally, there were six studies that utilized the HADS and/or the CES-D in the studies. 

#### 3.2.1. Anxiety and Depression Factors for QoL among Men Only Receiving Active Surveillance

In an examination of anxiety among men on AS, a correlational study indicated that 86% of men on AS had low general anxiety and 87% reported low levels of prostate cancer-specific anxiety [66]. Similarly, van den Bergh et al. (2009) examined decisional conflict among those undergoing AS wherein 92% of the participants were below the reference values for clinical depression, and 83% were below the reference values for clinical anxiety [43]. Other studies pertaining to anxiety and AS in the review found their populations to have values below the reference values for prostate cancer-specific anxiety [43,61]. In the review, two studies also reported a correlation between anxiety, depression, and disease-specific anxiety [43,44]. The decreased psychological issues from AS provide promise for increased QoL among individuals undergoing AS instead of radical treatments [43]. 

Simpson (2014) conducted a review of the literature on AS and the impact of prostate cancer on the patient′s decision for treatment [65]. The results from the article indicated that prior to electing AS as a treatment for prostate cancer, patients must be psychologically prepared to monitor the cancer [65]. A series of studies that examined the impact of AS on individuals reported relatively low levels of anxiety, a decrease in anxiety over time, and the anxiety levels remained stable while on AS [16,44,47,67]. In a qualitative study, prostate cancer-specific anxiety decreased from the time of diagnosis to 18 months after diagnosis [47]. Van den Bergh et al. (2010) reported low levels of anxiety among patients receiving AS during the first nine months [44]. Despite depression remaining consistent for the patients on AS, there was no increase in depression after nine months. Low levels of anxiety and depression among individuals treated with AS provide promising results for individuals to maintain or improve their QoL. Similar results were found in the qualitative study by Frydenberg et al. (2013), which revealed 91% of men did not have anxiety and 87% had low levels of prostate cancer-specific anxiety [68]. Overall, the men reported high levels of QoL and low levels of anxiety for localized prostate cancer. It is important to note that the men in the study received a significant amount of patient education, which could have contributed to the low anxiety levels and increased QoL. However, there were two predictors which were significant for predicting QoL, younger age and an inherent anxiety trait. 

There was an article that cited an increase in anxiety and depression among the men receiving AS as a treatment for prostate cancer. Watts et al. (2015) examined anxiety and depression in men undergoing AS for the treatment of prostate cancer. Clinical anxiety and depression were found to be 23% and 12.5%, respectively, among men receiving AS [42]. Previous data from men of the same age in the general population indicated rates of anxiety and depression to be 8% and 6%, respectively [42]. The results revealed rates of anxiety and depression that were twice as high among patients receiving AS compared to those without prostate cancer. For the most part, the articles in this review provided positive feedback for decreased rates of anxiety and depression among the men receiving AS for the treatment of prostate cancer. 

#### 3.2.2. Anxiety and Depression Factors for QoL among Active Surveillance and Other Forms of Treatments

Of the studies that compared the effects of AS to other forms of treatment for prostate cancer and the incidents of anxiety and depression, five examined anxiety and depression and one only examined anxiety. In addition, two studies found no significant differences in anxiety and depression levels between men who managed prostate cancer with only AS and those who underwent immediate radical treatment [35,41]. The articles within this integrative review examined the impact of AS and other forms of treatment for prostate cancer, such as, brachytherapy, radical prostatectomy, radical radiotherapy, and focal cryoablation [31,46] on QoL. Donovan et al. (2016) compared AS to radical prostatectomy and radical radiotherapy and found no significant differences in anxiety and depression between the groups [31]. Furthermore, Carter et al.′s (2015) systematic review regarding the psychological well-being and QoL among patients receiving AS and other forms of treatment for prostate cancer denoted no decrease in the psychological well-being of men receiving AS [69].

The results from the articles suggested that the majority of men on AS tended to report lower levels of anxiety and depression. However, the results were not inclusive of all the articles wherein some individuals on AS experienced higher levels of anxiety and depression compared with men receiving other forms of treatment for prostate cancer. The review cited articles that reported a contrast in results for higher levels of anxiety and depression [30,42,46]. De Cerqueira et al. (2015) conducted a study regarding anxiety and depression among prostate cancer survivors wherein participants on AS reported higher levels of anxiety and depression compared to participants that received brachytherapy and focal cryoablation [46]. Likewise, a large multisite study involving AS, QoL, and other forms of treatment in the United Kingdom indicated higher levels of anxiety and depression among the men who received AS [30]. 

The contrast in findings for higher levels of anxiety and depression among men receiving AS could be the result of psychological distress due to living with untreated cancer daily, which is one of possibly many reasons. The studies in the review highlight the need to examine the psychological issues encountered by men on AS. It is the examination of these issues which will provide additional data for identifying AS as a definitive, safe and beneficial treatment for prostate cancer. The results for this section in the review are presented in Table 2. 

## 4. Discussion

Research has shown that the determinants in the QoL of prostate cancer survivors were sexual, bladder, and bowel function [7,43,66]. It is not difficult to surmise that surgery and radiation treatments for prostate cancer have an adverse impact on sexual, bladder, and bowel function [7,46,49]. In essence, QoL is impacted by the treatment option for prostate cancer [46]. The findings from these articles address the sensitive issue of body image and the psychological impact it can have on men who encounter changes with their bladder function. An understanding of the impact of the various forms of treatment for prostate cancer can lead to interventions that meet the needs of men with decreased QoL. Interventions can also be developed that will maintain a sense of QoL among men receiving not only AS, but also other forms of treatment for prostate cancer. Furthermore, prostate cancer survivors must be aware of factors they will likely encounter if they choose curative treatments, such as radical prostatectomy, chemotherapy, and radiation treatment options.

An analysis of the articles included in the review indicated similar characteristics regarding the type of prostate cancer, use of questionnaires, and the study design. There was one stipulation that was consistent among the men who received AS for prostate cancer and it was the existence of low-risk prostate cancer. In essence, AS is not a curative form of treatment, which warrants its use only among localized low-risk prostate cancer. Despite some similarities in the characteristics among the articles, the varying types of studies caused difficulty in making comparisons. The concepts of QoL were similarly indicated by the factors of anxiety, depression, sexual, bladder, and bowel function. Within the articles in the current review, the concepts of QoL were predominately evaluated by questionnaires, such as, the EPIC, FACT-P, and the EORTC-QLQ-C30, except a qualitative study and one mixed methods study. The questionnaires were validated and were commonly found in the literature which evaluated QoL among individuals with prostate cancer. The disproportionately large number of quantitative articles in the current review did not allow for the evaluation of differences in QoL from questionnaires and interviews of prostate cancer patients receiving AS. 

Sexual functioning of prostate cancer survivors is inextricably intertwined with the prostate cancer survivor′s QoL. In terms of sexual function, the majority of studies found men undergoing AS to experience fewer issues in sexual function when compared to other treatments [6,29,31,32,45,50]. However, a single study found that patients on AS presented with a higher rate for decreased erectile function when compared to patients who underwent focal cryoablation and brachytherapy [46]. A cause for the results may be attributed to age, in which older men may experience increased erectile dysfunction.

In the current literature regarding AS patients, the vast majority of the studies found low levels of anxiety, prostate cancer-specific anxiety, and depression [43,66]. Conversely, there were studies that found no significant differences in anxiety and depression when compared with other treatment modalities [31,35,41]. Overall, three studies found that men on AS presented with higher anxiety and depression levels [30,42,46]. Despite the studies reporting higher anxiety and depression levels among their low-risk prostate cancer survivors, it is difficult to compare the results from these very different studies. De Cerqueira et al. (2015), unlike Lane et al. (2016), had a small sample size (30 vs. 1438) and did not involve multiple sites [30,46]. Watts et al. (2015) had a medium sample size of 426 and was also multicenter with comparisons to the general population [42]. Furthermore, different questionnaires were used in the studies to assess anxiety, the Beck Anxiety Inventory [46] and the Hospital Anxiety and Depression Scale [30,42]. The types of treatments were also different, which included a combination of AS, focal cryoablation, and brachytherapy [46] and AS, radical prostatectomy and external-beam conformal radiotherapy [30]. These findings, while limited in number, indicate the need for continued research regarding the mental health of low-risk prostate cancer survivors. It cannot be assumed that a diagnosis of low-risk prostate cancer excludes the possibility of mental distress among these men. 

The various themes identified in the review also included the comparison and evaluation of one or more factors associated with QoL and treatment for prostate cancer concurrently. It is apparent in a study published after the review (2017), which examined QoL among individuals with prostate cancer [70]. The results indicated that, after three years, the individuals who received a radical prostatectomy or external beam radiation had decreased sexual functioning and increased urinary incontinence compared to those who received AS. The invasive nature of surgery and the effects of radiation were far more detrimental, than AS, to the physiological functioning of men with prostate cancer. The decreased sexual functioning and increased urinary incontinence with the treatments other than AS matched the majority of results indicated in the current review. Similar results were reported in a two-year study, also published after the review that examined the impact of radical prostatectomy, radiation, brachytherapy, and AS on individuals with localized prostate cancer. In this study, radical prostatectomy had the highest rates of decreased sexual functioning and urinary incontinence compared to AS [71]. Additionally, radiation and brachytherapy treatment were commonly associated with urinary obstruction among the men in the study through two years. The large study in the United Kingdom, PROTEC T, also consisted of varying treatments and factors for QoL [30]. Symptoms from bowel and urinary issues were infrequent and older men had worse scores for urinary and sexual function. Only 1/5 of participants indicated problems with anxiety and depression. 

Active surveillance is recommended as a treatment option for low-risk prostate cancer. However, based on the literature, African Americans and other minority populations are not well represented in the current research. Due to the lack of diversity in the research, it is difficult to generalize these findings to all populations [35]. One of the articles in the review focused solely on the utilization of AS among African American men with prostate cancer [64]. The results indicated an uncertainty for implementing AS among this population. This gap calls for more research on QoL among African American prostate cancer patients. 

Overall, the results from the current review suggested that AS has a decreased negative impact on QoL when compared to other forms of treatment for prostate cancer. It could be that the decreased negative impact from AS on the QoL of prostate cancer survivors is due to the indications for the use of AS among low-risk prostate cancer. Men with low-risk prostate cancer may also perceive their health and diagnosis as less severe than an individual with advanced prostate cancer. A positive perception on health maybe a contributing factor into how they view their symptoms or even report the presence of symptoms. The thought of surgery, chemotherapy, and radiotherapy along with the recovery period and possible side effects can certainly be an area of concern and distress for any individual. Furthermore, men of older age tend to be diagnosed with prostate cancer and it is this older age that may lead to an improved outlook on life. The decreased side effects from AS indicate its use as an option for low-risk prostate cancer with a limited impact on QoL. There were only a few articles that indicated a negative impact on the QoL factors (sexual function, urinary continence, anxiety, and depression) [16,31,42,46,51,53] among men receiving AS while a limited number reported no change in the QoL factors for those that received AS [54,57]. Most of the articles reported declines in sexual functioning due to erectile dysfunction, bladder incontinence, bowel bother, anxiety, and depression among prostate cancer survivors who received a radical prostatectomy, radiation, or chemotherapy. The results corresponded with additional studies that evaluated the impact of various forms of treatment for prostate cancer on the individual′s QoL. 

## 5. Clinical Implications

The results from the study build upon the platform of research in which healthcare providers can offer AS as a beneficial treatment among their low-risk prostate cancer patients. Despite many prostate cancer patients who acquire knowledge of AS from the internet [63], healthcare providers must also be a pertinent source for education regarding AS. Healthcare providers will benefit by engaging in open and clear conversations regarding the side effects and pros and cons of treatment modalities for low-risk prostate cancer. Transparent communication is needed for what may be considered a sensitive topic, prostate cancer, and healthcare providers are in a prime role to lead the conversations. The dissemination of this information can strengthen patient and healthcare provider relations along with the possibility of encouraging communication among family members and friends for support. Psychosocial support is known to be an essential component of comprehensive care of cancer patients and should consequently be examined [41]. Based on the articles listed in this review, knowledge seems to be increasing regarding the the benefits and effects of AS within the context of QoL, whereas prostate cancer research among minority populations is clearly lacking. Additional research is needed to establish the impact of living with untreated prostate cancer on the QoL of the men. Further investigation of the PROMs of men on AS is a promising avenue for identifying factors relevant to their QoL. Through continued research, interventions can be developed to provide support, education, and interventions for maintaining or improving the QoL for men of various ethnic and racial backgrounds who undergo AS. 

## 6. Strengths and Limitations

The current integrative review strengthens the literature for studies focused on the impact of AS on the QoL for prostate cancer survivors. A focus on AS is a relevant topic in the field of prostate cancer given the increased exposure as a promising treatment for low-risk prostate cancer. The inclusion of various scholarly databases provided a diverse number of peer reviewed articles for inclusion in the review. Only empirical or theoretical articles were included in the review, which strengthened the acceptance of the credibility of the results from the articles included in the review. The use of two reviewers to collect, analyze, and organize the data allowed for a check and balance system for the inclusion of articles in the review. Overall, the review provides a breadth of knowledge on themes that many men find difficult to discuss. An awareness of the issues can ignite discussions and cause men to seek counseling or support for issues they once thought were taboo or too sensitive. 

The limitations of the integrative review are centered on studies that were not listed in the databases used in this review. Two reviewers were used to assist with a thorough review. However, the use of keywords may have also been a limitation in the review. Articles may have been excluded from the review due to the arrangement of the keywords used to search the databases. Furthermore, the inclusion timeframe for the studies excluded studies from this review. 

## 7. Conclusions

Through the use of a structured process, for conducting an integrative review, the factors of bladder, bowel, and sexual function, and anxiety and depression were identified as pertinent issues which impact the QoL among PCa survivors receiving AS. To date, there is not an integrative review focused solely on the concept of QoL among PCa survivors receiving AS. Within the 37 articles in the review, the majority used a quantitative design and mainly focused on QoL among the PCa survivors who received only AS as their form of treatment. A key attribute among the articles in the review was the commonality of low-risk PCa among the survivors that received AS. In general, there were higher levels of sexual, bladder, and bowel functioning and lower levels of anxiety and depression among those that received AS. Findings from the review were supported by similar studies regarding QoL among PCa survivors that received AS. However, there were a small number that indicated higher levels of anxiety and depression and lower levels of sexual, bladder, and bowel functioning. These findings could be attributed to the survivors knowingly living with PCa without receiving curative treatment. In essence, the review provided promising results for AS as a source of treatment that produced a positive impact on the PCa survivor’s QoL. The lack of racial and ethnic diversity among the participants in the studies and the variety of studies caused difficulty with making comparisons among the studies. However, the variety also provided insight into different methodologies to examine the concept of QoL among PCa survivors receiving AS as a form of treatment.

## Figures and Tables

**Table 1:** Sexual, bladder and bowel function and anxiety and depression among men utilizing active surveillance.

Citation and Source/Country	Purpose and Setting	Design, Methods and Sample	Race/Ethnicity	Reported Instruments	Key Findings	Strengths and Limitations
Acar et al., (2014)/ Amsterdam, the Netherlands [29]	Purpose: To investigate QoL after different treatment modalities for low-risk PCa with questionnaires. Setting: Amsterdam, the Netherlands.	Design: Quantitative, non-RCT. Methods: Questionnaires mailed or emailed, follow up every 6 months, 2004–2011. Statistics: Descriptives, Kruskal–Willis, Mann-Whitnal Grelyss, Spearman Correlations, and non-parametric Wilcox signed rank test. Sample: 144 out of 2615 eligible, low-risk PCa patients. Response rate: Not listed.	Race/Ethnicity: Not indicated.	Reported Instruments: QoL-EORTC-QLQC30; PCa QoL-EORTC-QLQ-CPR25; Sexual function-IIEF-15; Incontinence and QoL-ICIQ-SF.	Key Findings: AS patients had stable scores for physical QoL during follow-up. AS patients had lowest decrease (30%), in SF during follow-up brachytherapy (59%) and RALP (71%). Brachytherapy and RALP patients had decreased scores on different measures particularly, EF and incontinence.	Strengths: This study compared multiple treatment options and compared baseline QoL to QoL post-treatment. Limitations: Non-randomized set up causes a treatment selection bias, 80% of patients were referred to the tertiary oncology center, small sample size, comorbidities may have affected treatment choice, no data on QoL effects of delayed local treatment in the AS group were collected.
Banerji et al., (2015)/ United States [59]	Purpose: A comparison of HRQoL between patients managed by active surveillance or radiation therapy. Setting: Center for Prostate Disease Research (CPDR) Multicenter National Database, United States.	Design: Quantitative, non-RCT. Statistics: Descriptives and GEE. Methodology: A prospective cohort of patients at the Center for Prostate Disease Research Multicenter National Data base. Sample: Selected 77 (19%) of AS and 57 (14%) treated with EBRT of 410 low-risk PCa patients. Response rate: Not listed.	Race/Ethnicity: Large portion of African Americans indicated, but specific numbers not indicated.	Reported Instruments: HRQoL was measured with the EPIC and the SF36.	Key Findings: Majority treated with AS 77 (19%). More AA′s chose RT. Both groups had similar HRQoL scores at baseline. AS patients did not have declines in bowel or general physical HRQoL unlike patients who received the RT. Significant results for worse BF among RT patients at 1 year of follow-up *p* < 0.05 and 2 years *p* < 0.05. Statistically significant results for declines in BF and UB for radiation patients at 2 and 3 years follow up, *p* < 0.05. Statistically significant declines in overall physical health at 2 years, *p* < 0.01.	Strengths: The study compared the concept of HRQoL in AS and RT. Focused on the physical aspects of HRQoL, as opposed to the psychological aspects. Highlight the benefits of selecting AS based on reported HRQoL. Limitations: A comparison of HRQoL based on demographic data, such as, race, age, and education who have provided additional factors regarding their experiences with AS.
Bergman and Litwin (2012)/ United States [60]	Purpose: To examine existing literature regarding HRQoL among men on AS, instruments that measure HRQoL, and studies that examined HRQoL for men on AS. Setting: N/A.	Design: Literature review. Methodology: Literature review, examination of existing studies.	Race/Ethnicity: Not indicated.	Reported Instruments: HRQoL was examined through, retrospective, national, and experimental studies conducted in the United States.	Key Findings: Physicians should advise men with prostate cancer of the impact of treatment on QoL. Sexual dysfunction and anxiety were present in men receiving AS. A 5-year follow-up indicated increased erectile dysfunction and urinary leakage among patients receiving AS compared to curative treatments. AS patients had less urinary obstruction. After 5 years, scores for BF, anxiety, depression, and general HRQoL were similar.	Strengths: This review of literature on HRQOL, encompasses, the concept of HRQOL, as well as, instruments utilized to examine HRQOL, and various studies which examined HRQOL. Limitations: The information would be easier to discern if it was presented in a table format.
Braun et al., (2014)/ New York, New York and Herne, Germany [53]	Purpose: To explore the hypothesis that serial biopsies can lead to reduced EF in men undergoing AS. Setting: New York, US and Herne, Germany.	Design: Quantitative, non-RCT. Methods: Questionnaires given to participants at scheduled clinic visits. Follow-up annually for 4 years. Statistics: Locally weighted scatterplot smoothing. Sample: 342 men on AS between 2000–2009. Response rate: Not listed.	Race/Ethnicity: indicated.	Reported Instruments: QoL and EF measured with the PHRQoL and 6 questions from the IIEF.	Key Findings: Small decrease in EF over a period in men undergoing AS. AS biopsies did not have a large impact on EF.	Strengths: Possible changes in EF were evaluated longitudinally. Limitations: Change in EF was not measured within days or weeks after biopsies, there was no control group to distinguish between the effects of aging alone versus those of aging and repeat biopsies.
de Cerqueira et al., (2015)/ Sao Paulo, Brazil [46]	Purpose: To identify the burden of three different protocol-based treatment options. Setting: Sao Paulo, Brazil.	Design: Quantitative, non-RCT. Methods: Questionnaires were given to participants and follow-up in 1 year. Statistics: Descriptives, Spearman correlations, and ANOVA. Sample: Invited 130, 100 excluded, final of 30 with very low risk PCa. Response rate: None listed.	Race/Ethnicity: Not indicated.	Reported Instruments: HRQoL measured by the SF-36, EF measured by 5 questions from the IIEF voiding functions measured by the IPSS, anxiety was measured by the BAI, hopelessness was measured by the BHS, depression was measured by the BDI.	Key Findings Patients who opted for AS reported higher levels of hopelessness and worse general health perceptions when compared to BT and FC.	Strengths: This study offered a comprehensive assessment of low-toxicity prostate cancer therapies and used many different standardized instruments. Limitations: Very small sample size and age difference is a possible confounder.
Donovan et al., (2016)/ United Kingdom [31]	Purpose: To investigate the effects of active monitoring, RP, and radical radiotherapy with hormones on patient-reported outcomes in the Prostate Testing for Cancer and Treatment (ProtecT) trial. Setting: United Kingdom.	Design: Quantitative, randomized. Methods: Questionnaires given to participants. Follow-up at 6 and 12 months over 6 years. Statistics: Descriptives, logistic models, two-level linear models. Sample: 2896 identified with PCa in 1999–2009, 1643 were randomized. Response rate: 85%.	Race/Ethnicity: Not indicated.	Reported Instruments: UF was measured by the ICIQ; SF and BF was measured by the EPIC, general health was measured by the SF-12, anxiety and depression were measured by the HADS, cancer-related QoL was measured by the EORTC QLQ-C30.	Key Findings: AS had little effect on urinary continence; EF decreased from year to year; BF and BB remained the same.	Strengths: Long-term study with a large sample size. Limitations: The forms of treatment for prostate cancer were not consistent in the study and men switched treatments. Lack of diversity.
Ercole et al., (2014)/ Cleveland, Ohio [50]	Purpose: Analyze voiding, BF, SF, urinary incontinence, and physical/emotional functioning among patients managed by AS, RP, and BT. Setting: Cleveland, Ohio.	Design: Quantitative, non-RCT. Methods: Instrument was given to patients. Follow-up at 6 months and 12 months over 2 years between 2007 and 2013. Sample: 590 patients with PCa. Response rate: None listed.	Race/Ethnicity: Not indicated.	Reported Instruments: The domains were measured by Qual Life Res 2000.	Key Findings: All of the domains regarding QoL for patients on AS were stable over 1-2 years for UF and BF. AS was significantly better than BT. SF and incontinence for AS was significantly better than RP.	Strengths: Use of instrument for self-reported functional outcomes and time was treated as categorical instead of continuous to reflect possible non-linear time trend. Limitations: Possible selection bias, making results less generalizable.
Hayes et al., (2010)/ United States [52]	Purpose: Examine QoL risks and benefits among patients receiving AS and other initial therapies as a treatment for PCa.	Design: Quantitative, non-RCT Methodology: Simulation decision model analysis utilizing BT, intensity-modulated radiation therapy, RP or AS. Statistics: Descriptives, State transition model, 1-way multiway sensitivity analyses with a 1-time input. Sample. 500 samples consisting of 100,000 individual trials, however a definite sample size was not specified. Hypothetical groups of men aged 65 years and older diagnosed with localized low risk PCa. Response rate: Not listed.	Race/Ethnicity: Not indicated.	Reported Instruments: A model was utilized based on previous sources from the literature which included the components of annual probabilities, base case estimates, and a range used in sensitivity analysis.	Key Findings: Men over 65 which received AS were expected to live an additional 6 months of quality of life age expectancy. Despite high risk for death from PCa, the men on AS still maintained the highest quality adjusted life-years.	Strengths: An examination of QoL based on adjusted life-years. The study is a first to use decision analysis and a model which used previous literature to determine probabilities and utilities, which was innovative. Limitations: The participants were hypothetical and there was only one age for the men, 65 years. The model only included what was reflected in the literature. There was not a clear sample size provided for the hypothetical patients. Results indicated there were.
Hegarty et al., (2008)/ United States and Ireland [54]	Purpose: The purpose is to explore uncertainty and QoL among men 65 and over undergoing AS as a treatment for prostate cancer in America and Ireland.	Design: Quantitative, non-RCT. Questionnaires mailed, no follow-up. Statistics: Descriptives, Cronbach alpha score. Sample: 92 questionnaires mailed to patients in Ireland, 58 returned, only 2 agreed to participate. In America, 27 agreed to participate for a total of 29.	Race/Ethnicity: Not indicated.	Reported Instruments: Uncertainty measured by the MUIS-C, QoL was measured using the Quality of Life Index, the Cancer Version for QoL, the UCLA-PCI for measuring six domains (urinary function, urinary bother, bowel function, bowel bother, sexual function, and sexual bother) related to QoL among PCa survivors.	Key Findings: Uncertainty was higher for men in America undergoing AS. The HRQOL scores were similar among patients in Ireland and America. The men in Ireland had lower mean and social functioning compared to men in America. Men in Ireland also reported more energy and improved general health. The men in Ireland also indicated they had more issues with UF and less concern with sexual issues.	Strengths: There was a comparison of AS treatment for PCa among men in Ireland and America. Limitations: A small sample size and it was a convenience sample. Lack of racial diversity among the samples.
Jeldres et al., (2015)/ United States [6]	Purpose: To assess the impact of PCa management strategy on disease-specific and general HRQoL outcomes over time. Setting: Sites included Madigan Army Medical Center (Tacoma, Wash), Naval Medical Center (San Diego, Calif), Virginia Mason (Seattle, Wash), and Walter Reed National Military Medical Center (Bethesda, Md).	Design: Quantitative, non-RCT Methods: Questionnaires were administered to participants. Annual follow-up for 3 years. Statistics: Descriptives, Welch tests, chi-square, Cochran–Armitage, GEE. Clinically meaningful was established as a 0.5 difference in the standard deviations among the baseline scores in each cohort. Sample: 745 eligible 305 participated which were enrolled in the Center for Prostate Disease Research (CPDR) Multicenter National Database.	Race/Ethnicity: White: 224 African American: 58 Hispanic: 9 Asian: 12 Unknown: 2.	Reported Instruments: Function and bother for urinary, sexual, bowel, and hormone domains were evaluated by the EPIC and mental components were measured by the SF-36.	Key Findings: In the AS cohort, there were no statistically significant or clinically meaningful declines in QoL. The RP cohort experienced clinically meaningful and statistically significant declines in SF, sexual bother, and UF scores that persisted for 3 years.	Strengths: This study is one of the first to report on longitudinal HRQoL in a carefully defined, prospective cohort of patients who underwent AS; the multidisciplinary approach increased study strength; racial diversity; use of qualified HRQoL metrics. Limitations: Participants were self-selected and not randomized into treatment groups, so it is possible that the patients who chose AS were less anxious than those who chose treatment, small AS sample, generalizability of our findings may also be limited given our strict eligibility criteria and unique cohort features (example: most of the subjects were military health care beneficiaries).
Kasperzyk et al., (2011)/ Boston, Massachusetts [32]	Purpose: To examine patient reported outcomes among patients with PCa treated with watchful waiting in a nationwide cohort. Setting: Multiregional, American, community-based setting.	Design: Quantitative, non-RCT Methods: Questionnaires administered to patients. Only largest follow-up reported, which was at 7.6 years. Statistics: Descriptives, Cox proportional, Hazards regression, logistic regression, *t*-tests, chi-square, Fisher exact tests, Wilcox rank-sum tests, D′Amico criteria. Sample: Invited 3313 invited, 1366 participated at baseline, 1230 final sample. Patients from the Physicians′ Health Study.	Race/Ethnicity: White: 95.6% Black: 0.9% Asian: 1.4% Other: 2.1%	Reported Instruments: QOL questions included items from the UCLA-PCI and the EPIC.	Key Findings: When watchful waiting and AS was compared to immediate treatment, patients who underwent watchful waiting had lower urinary incontinence and impotence but more common obstructive urinary symptoms.	Strengths: Chi-square test, Wald test, and logistic regression used. Limitations: Baseline information not available, recall bias, could not differentiate between AS and watchful waiting, could not examine lethal PCa as an endpoint.
Kazer et al., (2011)/ United States [55]	Purpose: Examine the impact of the intervention, Alive and Well on decreasing uncertainty, improving self-management, self-efficacy and QoL in men undergoing AS for prostate cancer. Setting: Two urological practices at academic institutions in the Northeastern, United States.	Design: Quantitative, non-RCT, single-subject design. Questionnaires were completed online, and participants were informed to visit the Alive and Well website as an intervention Follow-up at weeks 5 and 10. Statistics: Pearson correlations. Sample: Started with 20 participants and final was 9. Response rate: Attrition rate listed as 33%.	Race/Ethnicity: White: 9.	Reported Instruments: Self efficacy scale adapted developed by Lorig et al. (1996), Uncertainty measured by the MUIS-C, QoL was measured with the UCLA-PCI.	Key Findings: There were improvements in 8 of the 12 subscales for QoL at T2 when compared to baseline. QoL scores went back to baseline at T3.	Strengths: The novelty of a solely online design. A study which is focused on identifying factors which impact participants QoL while undergoing AS. Limitations: Attrition rate of 33% yielded a small sample. The use of solely online instruments among an older population may have caused attrition.
Kazer et al., (2011)/ United States [63]	Purpose: Conduct focus groups to explore the psychosocial and educational needs of men undergoing AS for prostate cancer. Setting: United States.	Design: Qualitative Methodology: Structured interview questions were provided to the focus groups, which lasted approximately one hour. A male researcher conducted the interviews, which were audio recorded. Sample: 7 participants participated in two focus groups.	Race/Ethnicity: White: 7.	Reported Instruments: A set of focus group questions was developed for the participants.	Key Findings: The following themes were identified from the study: sources of information, disease monitoring/vigilance, myths/misinformation/frequently asked questions, and Health promotion and taking charge. Participants turned to the internet to obtain information, the men made life style changes after the diagnosis of prostate cancer.	Strengths: Qualitative study to explore the impact of AS on HRQOL. Two researchers examined the data. A male researcher provided the questions to the participants. Limitations: There was a lack of racial/ethnic diversity among the participants.
Lane et al., (2016)/ Nine cities in the United Kingdom [30]	Purpose: To examine the patient reported outcomes of men diagnosed with and receiving treatment for localized PCa.	Design: Quantitative, randomization Methods: Paper questionnaires were distributed to patients at clinics during their initial prostate specific antigen screening and biopsy. Some participants were randomized to complete the questionnaires by mail at 6 months and yearly for 10 years. Sample: 2417 identified, but 1438 completed the questionnaires and received the biopsies. Response rates: Not listed.	Race/Ethnicity: 99% White.	Reported Instruments: QoL measured by the EQ-5D-3L, Urinary and sexual functions except for hormonal domains measured by the EPIC, incontinence measured by the ICIQ-UI, l Continence measured by the ICSmaleSF, anxiety and depression measured by the HADS, the SF-12 was used to measure general mental and physical health.	Key Findings: AS was second to RT as the most common form of treatment. The lowest scores for issues with SF and problems was among the AS group. Participants receiving AS had higher anxiety and depression, health utility, mental, and physical health scores. There was no difference among the groups for significant problems with erectile dysfunction.	Strengths: Large multi-site study that utilized randomization for completing questionnaires. High completion rate of the questionnaires. Limitations: Some participants only completed the baseline questionnaires which could not be compared to subsequent follow up results from other participants. The baseline questionnaire was completed at the initial biopsy, which may have been a stressful time for participants. Results cannot be generalized to non-white individuals.
Lokman et al., (2013)/ Helsinki, Finland [56]	Purpose: To investigate the effect of AS protocol on HRQoL, erectile function and UF in low-risk PCa patients. Setting: Helsinki University Central Hospital.	Design: Quantitative, non-randomized. Methods: Questionnaires were given to patients annually for 3 years. Statistics: Descriptives, paired *t*-tests. Sample: 224 patients enrolled in the Finnish arm of the Prostate Cancer Research. International: AS (PRIAS) study. Response rate: Not listed.	Race/Ethnicity: Not indicated.	Reported Instruments: General health measured by the RAND 36, EF measured by the IIEF-5 and PCa symptoms measured by the IPSS.	Key Findings: Using a generic QoL questionnaire (RAND 36), no deterioration of QoL was apparent after 3 years of follow up in a prospective AS cohort. No detrimental effect on EF.	Strengths: Prospective design and use of standardized questionnaires, long term follow up and baseline scores were obtained. Limitations: Lack of randomization when selecting patient population.
Parker et al., (2016)/ Houston, Texas [16]	Purpose: To evaluate prospectively the associations between illness uncertainty, anxiety, fear of progression and general and disease-specific QoL in men with favorable-risk PCa undergoing AS. Setting: Houston, Texas. QoL outcomes for men who discontinued AS.	Design: Quantitative, non-RCT. Methods: Participants completed questionnaires at the time of enrollment and every 6 months for up to 30 months. Statistics: Descriptives, mixed models, and compound symmetry covariance structure. Sample: 180 men during 2006–2012 with favorable low-risk PCa. Response rate: Not listed.	Race/Ethnicity: White: 86% Black: 6.7% Hispanic: 6.1% Asian: 1.1%.	Questionnaires assessed illness uncertainty, anxiety, prostate-specific QoL using the EPIC, SF-12, MUIS, and the STAI.	Key findings: QoL was stable after a 2.5-year follow-up, which indicated a decrease in SF scores. An increased PSA score was associated with a decreased urinary and bowel score. As illness uncertainty increased, urinary, bowel, sexual, hormonal, and satisfaction scores decreased. An increase in anxiety predicted lower urinary, bowel, sexual, hormonal, and satisfaction scores.	Strengths: This study is one of the largest prospective studies to examine QoL, the influence of psychosocial factors on QoL, and fear of disease progression for men who are on AS. This study controlled for demographic and cancer-related variables. Limitations: The cohort at this specialized cancer center may be different from other cohorts; AS criteria was different from other trials; the sample was 86% white, so it may be difficult to generalize the results to other races and ethnicities; this study did not assess psychosocial and QoL outcomes for men who discontinued AS.
Pham et al., (2014)/ Seattle, Washington [51]	Purpose: To specifically assess the HRQoL impact of AS compared to a control group of men who had undergone negative PNB. Setting: Multidisciplinary clinic.	Design: Quantitative, non-RCT, prospective cohort study. Follow-up annually for 2 years. Methods: Questionnaires were given to patients at baseline and PNB. Statistics: Descriptives and univariate predictor analysis. Sample: 326 (223 PNB patients and 103 AS patients from the Center for Prostate Disease Research (CPDR) multi-center national database during 2007–2012. Response rate: Not listed.	Race/Ethnicity: White: 326 Non-caucasian:133.	Reported Instruments: The SF-36 measured physical and mental health and the EPIC measured sexual, urinary, and bowel function and bother.	Key Findings: AS had statistically significant declines in SF at 1 and 2 years and UF at 2 years when compared to PNB negative patients. Importantly, there were no HRQoL differences in BF, physical health or mental health between the 2 groups.	Strengths: Use of a control group. Limitations: Relatively small sample size.
Pham et al., (2016)/ [58]	Purpose: Evaluate HRQoL outcomes in men on AS compared to men followed negative PNB, non-cancer. Setting: Various institutions.	Design: Quantitative, randomization, prospective study. Methods: Questionnaires administered annually for 3 years at clinic visits for PNB. were given to patients. Statistics: Welch′s *t*-test, chi-square, GEE, Fisher′s exact test, Cochran-Armitage trend tests. Sample: 1204 eligible, 787 had PCa and 411 had low-risk PCa and 89 on AS. Response rates: Non- cancer (61%) and AS (67%).	Race/Ethnicity: White: 343 African American: 79 Hispanic: 19 Asian: 47 Other: 14 Unknown: 7.	Reported Instruments: HRQoL was assessed using the SF-36 and EPIC questionnaires.	Key Findings: AS patients reported higher scores at baseline and 1 year for UF and UB, BB, hormonal bother, physical component summary, role-physical, bodily pain and social functioning subscales. Projected trends over time indicated decreased UF, BF and bodily pain among the men receiving AS.	Strengths: Use of randomization. This study is the first to compare prospective, longitudinal HRQoL in patients who underwent AS to that of subjects with a negative PNB. Limitations: Absence of baseline data.
Punnen et al., (2013)/ San Francisco, California [49]	Purpose: To assess long-term QoL in men with PCa using a longitudinal, nationwide, PCa registry. Setting: Nationwide, United States.	Design: Quantitative, non-RCT. Methods: Questionnaires administered to patients yearly for follow-up over 10 years. Statistics: Descriptives and repeated measures mixed model regression analysis. Sample: Cohort consisted of 3777 in which 2018 (60%) underwent RP, 686 (20%) underwent BT, 392 (12%) underwent external beam RT, 197 (6%) underwent primary androgen deprivation therapy and 84 (2%) underwent AS or watchful waiting from the Cancer of the Prostate Strategic Urologic Research Endeavor (CaPSURE) database. Response rate: Not listed.	Race/Ethnicity: Not indicated.	Reported Instruments: QoL was assessed using the UCLA-PCI and the SF-36.	Key Findings: Most had initial declines in HRQoL in the first 2 years after treatment. Almost no change in years 3 through 10.	Strengths: Longitudinal study from a nationwide PCa database. Limitations: Lack of randomization.
Punnen et al., (2013)/ San Francisco, California [41]	Purpose: To assess the presence of depression, anxiety, and distress among patients who received AS and RP and the impact on urinary and sexual QoL at baseline and follow-up. Setting: University of California, San Francisco (UCSF) Dept. of Urology.	Design: Quantitative, non-RCT. Methods: Questionnaires from the institutional Urologic Oncology Database. Follow-up at 1 and 3 years from baseline. Sample: 864 invited, 679 participated AS (122) or RP (557). Response rate: 77% baseline reported.	Race/Ethnicity: White: 622 Asian/Pacific Islander: 31 African American: 9 Latino: 8 Mixed: 4 Other: 4 Native American: 1.	Reported Instruments: Depressive symptoms were assessed using the PHQ-9, anxiety symptoms were measured using the GAD-7, distress was ascertained using the DT, erectile dysfunction measured by the SHIM, UB, sexual bother assessed by the EPIC-26, urinary issues assed by the IPSS.	Key Findings: Similar rates of depression, anxiety and distress among patients receiving AS or RP over time. Higher levels of depression or anxiety were associated with worse SF and bother, while elevated levels of distress were associated with UF on follow-up.	Strengths: Use of several measures for psychological and physiological distress. Limitations: Did not assess for past medical history of depression or anxiety, men might be less likely to endorse mental health symptoms on an online survey; potential bias.
Seiler et al., (2012)/ Switzerland [62]	Purpose: To determine the level of anxiety and HRQoL among men receiving AS and their partners. Setting: Switzerland.	Design: Quantitative retrospective design. Methods: Men were recruited from the European Randomized Study of Screening for Prostate Cancer to complete the questionnaires. Follow up at 17, 32, 59, and 136 months, data collected between February and August 2010. Statistics: Wilcox test, ANOVA, binary logistic regression Sample: 283 invited. There were a total of 133 (*n* = 266) couples in the study. Response rate: 46.9%.	Race/Ethnicity: Not indicated.	Reported Instruments: Anxiety and depression measured with the HADS and the MAX-PC, aspects of QoL measured with the EORTC QLQ-30.	Key Findings: Low anxiety and distress levels were reported by patients and their partners. The distress level for patients and their partners did not reach a clinically relevant level. The partners had higher HRQoL scores for the domains of pain, global health status, physical and emotional functioning, fatigue, dyspnea, insomnia, and constipation. There was an association among elevated anxiety levels in the partners and the length of time on AS, lower general health status of the partners, and decreased emotional functioning.	Strengths: The study examined QoL among patients on AS for PCa as well as their partners. Comparisons were made to examine level of anxiety among the men and their partners. Limitations: The study was from a single-center in a small country. There was no control group and some of the data were based on recall, which may decrease the accuracy of the information.
Silberstein et al., (2014)/ New Orleans, Louisiana [64]	Purpose: A review of the literature on AS among AA men due to AA remaining at greater risk of disease progression. Setting: New Orleans, Louisiana.	Design: A review of the literature. Methods: Conducted utilizing the electronic databases Medline (PubMed). The inclusion dates were articles published through March 2014. The articles were categorized into the following groups retrospective studies, prospective observational studies, and prospective randomized trials. Sample: A specific number of articles was not indicated in the review of literature.	Race/Ethnicity: Retrospective studies-African American: 256 out of 1801, Prospective observational- African American: 125, Prospective randomized trials-American: 30% White 70%.	Reported Instruments: There were no instruments due to the study being a review of literature.	Key Findings: The majority of studies were small without any reported power, from a single institution and the retrospective studies had cohorts which lacked consistent findings regarding the safety of AS for AA men. The pathological features for AA men with low risk prostate cancer tended to be worse than those for White men. There may be a need for better imaging among AA men due to the location and size of the tumor. AA men on AS may have increased progression of prostate cancer or choose to forego AS a treatment. AS can still be a viable option of treatment for this high-risk population.	Strengths: The article is a review of literature which dates back to the oldest electronic source through 2014. Limitations: A limitation is that only one electronic database was utilized in the study. The inclusion of additional databases could have produced an abundance of articles for the manuscripts. The review did not focus on the type of instruments in the studies.
Simpson (2014)/ Canterbury, England [65]	Purpose: (1) A rapid literature search regarding the psychological impact of AS for treatment of patients with PCa. (2) Examining who assumes responsibility for the patient follow up on AS. Setting: Canterbury, England.	Design: A review of the literature utilizing, CINAHL plus with full text online, MEDLINE, text books, and journal articles. The article focused on the concepts of prostate cancer staging and grading, AS guidelines for practice, and psychological impact. Sample: A specific sample size was not indicated.	Race/Ethnicity: Not indicated.	Reported instruments: The SF36, Health Status Survey, measures HRQoL, the perceived stress scale, the Sexual Function Score and Prostate Cancer Index. The Memorial Anxiety Scale for Prostate Cancer (MAX-PC), IIEF-5 and the IPSS assessed stress and functional issues.	Key Findings: QoL determined from PCa treatments. Patients should only engage in AS if they are psychologically prepared to accept the monitoring of their PCa. Close monitoring of patients on AS for PCa is needed to further assess their psychological well-being. Trained nurse clinical specialist in the area of communications can be utilized to follow up with the psychological assessments of patients receiving AS. Education is a key component for patients receiving AS for PCa.	Strengths: The literature review appeared to be exhaustive in its use of electronic databases and the author′s own personal resources. Limitations: There was a lack of notation regarding the process for identifying, including, and excluding sources in the literature review.
Vasarainen et al., (2011)/ Helsinki, Finland [57]	Purpose: To analyze longitudinal changes in general, mental and physical QoL and urinary and erectile function in patients with low-risk PCa on AS. Setting: Finland.	Design: Quantitative, non-RCT. Methods: Questionnaires completed at the start of AS and at the first biopsy. Follow-up at 1 year. Statistics: A paired t -test, Correlation analysis, Pearson chi-square. Sample: 124 eligible and final sample of 80 from the Finnish arm of the Prostate Cancer Research. International: Active Surveillance (PRIAS) study. Response rate: 85% baseline questionnaires and 94% completed baseline and follow-up.	Race/Ethnicity: Not indicated.	Reported Instruments: General HRQL was assessed with the RAND 36-Item Health Survey (RAND-36), EF assessed with the IIEF-5, and urinary symptoms with the IPSS.	Key Findings: The low-risk PCa patients who received AS did not experience negative impacts on their QoL. UF and EF did not produce statistically significant changes. At one-year follow-up there were no differences in the QoL factors of mental and physical changes. Among the 8 QoL dimensions, only physical role improved and was statistically significant. Patients receiving AS experienced significantly better general mental and physical HRQL than the Finnish male population.	Strengths: Prospective design and various questionnaires. Limitations: Lack of randomization.
van den Bergh et al., (2012)/ The Netherlands [45]	Purpose: To compare SF of men with localized PCa on AS with similar patients who received radical therapy. Setting: Erasmus University Medical Centre and of participating local hospitals.	Design: Quantitative, no-RCT. Methods: Questionnaires were administered at the time of diagnosis or at the time of their treatment. Follow was done at 6 and 18 months for the AS group, 12 months for the RP and RT group. Statistics: Multivariable analysis, independent sample *t*-tests, and linear regression analysis. Sample: A total of 266 (AS = 129; RP = 67; RT = 70) patients with localized PCa. Response rate: Only listed for AS patients, 129 completed baseline questionnaires and 60% completed at follow-up.	Race/Ethnicity: Not indicated.	Reported Instruments: Questionnaires contained 10 items on SF, the mental and physical component summary from the SF-12, depression was assessed with the CES-D and general anxiety was measured with the STAI-6.	Key Findings: Fewer men undergoing AS were less sexually active due to EF when compared with patients who received combined treatment. more men from the active Sexual activity was increased among the men AS group along with decreased issues with EF.	Strengths: Use of Comparison of treatment groups for PCa. Limitations: Not randomized, no baseline measurement of SF.
Wilcox et al., (2014)/ Gosford, Australia [61]	Purpose: To assess anxiety QoL and understanding of AS in a cohort of patients enrolled in AS for PCa. Setting: Gosford Hospital and Gosford Private Hospital.	Design: Quantitative, non-RCT. Methods: Patients were mailed questionnaires to complete and return. No follow-up. Sample: 61 were invited to participate and 47 responded. Response rate: 77%.	Race/Ethnicity: Not indicated.	Reported Instruments: SF was assessed using the IIEF-5, voiding using the IPSS and the MAX-PC measured of PCa specific anxiety.	Key Findings: There were low levels of anxiety among the patients on AS and there was no difference in their QoL. Patients on AS did not experience difficulties with UF or EF while on AS.	Strengths: This study represents one of the first Australian investigations on HRQL and anxiety in men on AS of prostate cancer. Limitations: Small sample size.

AS = Active surveillance; BAI = Beck Anxiety Inventory; BB = Bowel bother; BDI = Beck Depression Inventory; BF = Bowel function; BHS = Beck Hopelessness Scale; Brachytherapy = BT; CES-D = Center for Epidemiologic Studies Depression scale; DT = Distress Thermometer; EF = Erectile function; EORTSQLQ-C30 = European Organization for Research and Treatment of Cancer Quality of Life Questionnaire Core 30; EORTC-QLQ-PR25 = European Organization for Research and Treatment of Cancer Quality of Life Questionnaire Prostate Module; EPIC = Expanded Prostate Cancer Index Composite; EPIC-26 = Expanded Prostate Cancer Index Composite Short Form; EQ-5D-3L = EuroQol quality-of-life survey; FC = Focal cryoablation; GEE = Generalized estimating equations; HRQoL = Health related quality of life; HADS = Hospital Anxiety and Depression Scale; ICIQ-SF = International Consultation on Incontinence Questionnaire Short Form; ICIQ = International Consultation on Incontinence Questionnaire; ICSmalesSF = International Continence Society short-form male survey; IIEF-15 = International Index of Erectile Function; IPSS = International Prostate Symptom Score; MAX-PC = Memorial Anxiety Scale for Prostate Cancer; SF36 = Medical Outcomes Study Short Form; MUIS-C = Mishcel Uncertainty form; PCa = Prostate; PHRQoL = Prostate Health Related Quality of Life; PNB = Prostate needle biopsy; QoL = Quality of life; RALP = Robot-assisted laparoscopic prostatectomy: RP = Radical prostatectomy; RT = Radiation therapy; SF = Sexual function SHIM = e Sexual Health Inventory for Men; STAI = State-Trait Anxiety Inventory for Adults; UB = Urinary bother; UCLA-PCI = UCLA Prostate Cancer Index; UF = Urinary function.

**Table 2:** Anxiety and depression among men utilizing active surveillance.

Citation and Source/Country	Purpose and Setting	Design, Methods and Sample	Race/Ethnicity	Reported Instruments	Key Findings	Strengths and Limitations
Alvisi et al., (2013)/ Milan, Italy [67]	Purpose: To investigate the changes in HRQoL and adjustment to the first 2 years on AS. Setting: PRIAS database.	Design: Quantitative, non-RCT. Methods: Questionnaires completed at enrollment, 10 after diagnostic biopsy, 12 months after re-biopsy and 24 months. Statistics: Repeated measure analyses of variance were performed to test changes over time and Bonferroni correction. Sample: 208 patients. Response rate: At 10 months 156 completed questionnaires, 12 months had 109 completed questionnaires, and at 24 months 62 completed questionnaires.	Race/Ethnicity: Not indicated.	Reported Instruments: HRQoL domains were measured by the FACT-P; strategies of coping with cancer were measured by the Mini-MAC.	Key Findings: Patients on AS reported high levels of physical and psychological wellbeing throughout the first two years. QoL was not impaired by the idea of living with an untreated cancer.	Strengths: Long term study. Limitations: Patients selected may have already had low anxiety.
Anderson et al., (2014)/ Victoria, Australia [66]	Purpose: Describe a range of anxieties among men on AS for PCa and determine which of these anxieties predicted HRQOL. Setting: Cabrini Health in Victoria, Australia.	Design: Quantitative, non-RCT. Methods: Questionnaire were mailed to eligible patients. No follow-up. Statistics: Descriptives, Pearson′s correlations, and hierarchical regression. Sample: 260 men on AS were invited. 86 returned the questionnaires. Response rate: 33%	Race/Ethnicity: Not indicated.	Reported instruments: Psychological measures including the HADS, STAI, MAX-PC and the FACT-P anxiety; fear of reoccurrence; sociodemographic information.	Key findings: Men in the study had normal levels of general anxiety and illness-specific anxiety and high PCa related HRQoL. Age, trait anxiety and fear of recurrence were significant predictors of PCa related HRQoL.	Strengths: Multiple psychological measures were included to represent a wide range of anxieties. Limitations: results should be considered in the context of sample characteristics, the study had a correlational design (which cannot establish cause-effect relationship), use of only one facility.
Bellardita et al., (2012)/ Milan, Italy [39]	Purpose: Presentation of preliminary results for the observation of HRQoL, adjustment to disease and mental health of some patients from the PRIAS: AS study. Setting: Milan, Italy.	Design: Quantitative, non-RCT. Methods: The PRIAS database used several studies, the current study did not include follow-up information. Statistics: Descriptives. Sample: 70 participants between September 2007 and 2009 from the PRIAS cohort.	Race/Ethnicity: Not indicated.	Reported instruments: HRQoL was measured using the FACT-P, the Mini-MAC, assessed how patients cope with cancer; the SCL-90 report assessed patient′s mental health status.	Key Findings: Very high scores for HRQoL and no issues with adjusting to cancer. AS seemed to preserve QoL without adding any mental health issues.	Strengths: Use of longitudinal database comprised of data from 100 medical centers in 17 countries. Limitations: Possible selection bias.
Burnet et al., (2007)/ London, UK [35]	Purpose: To investigate anxiety and depression in patients with localized PCa managed by AS and those that selected immediate treatment. Setting: London, UK.	Design: Quantitative, non-RCT, cross-sectional. Methods: Participants received questionnaires in outpatient clinics. No follow-up. Statistics: Descriptives, One- way ANOVA, Least significant difference, chi square, and Bi-serial correlations. Sample: 764 patients were identified, 493 had early stage PCa, 353 completed the HADS, 24 excluded based on responses, final count of 329. Response rate: 72 % completed the HADS out of the 353.	Race/Ethnicity: White: 307 Other: 22.	Reported instruments: Anxiety and depression assessed by the HADS.	Key Findings: AS, compared to immediate treatment, did not cause an increase in anxiety or depression.	Strengths: Comparison of AS and immediate treatment for psychological distress. Limitations: Lack of randomization and use of only one measure for anxiety and depression.
Carter et al., (2015)/ Australia [69]	Purpose: (1) Examine the impact of AS on the patient′s psychological well-being and QoL. (2) Comparison of AS with active treatments for impact on psychological health. Setting: Australia.	Design: Systematic review. Methodology: the PRISMA guidelines were utilized to conduct the review. The following data bases were searched: Medline, PsycINFO, EMBASE, CINHAL, Web of Science, Cochrane Library and Scopus. Inclusion criteria were articles published January 2000–2014.	Race/Ethnicity: Not indicated.	Reported Instruments: The following measures were reported in the systematic review: HADS (8–10 borderline and >10 clinical), MAX-PC (P27), EORTC QLQ-C30, STAI-6 (>44), CES-D (P16), DCS (>37.5), STAI E, SF-36, UCLA-PCI, QLI- MUIS, SCL-90, FACT-P, and Mini-MAC.	Key Findings: There were 34 articles that met the inclusion criteria, 24 observational, 8 RCTs, and 2 interventional studies. No adverse impact from AS on psychological well-being. and no differences in psychological wellbeing compared to active treatments.	Strengths: Rigor of using a systematic review and the PRISMA guidelines. Limitations: Only Western countries and English language were included in the study. No consideration for men who started on AS and later chose an active treatment. Longitudinal studies did not have final results.
Frydenberg et al., (2013)/ Melbourne, Australia [68]	Purpose: To describe the anxieties among men on AS, and which anxieties predicted HRQoL. Setting: Melbourne, Australia.	Design: Quantitative, non-RCT. Methods: Questionnaires distributed to patients at a urologist′s office. No follow-up. Statistics: Descriptives. Sample: 265 men from a urologist′s AS database identified. 104 participated in the study. Response rate: Not indicated.	Race/Ethnicity: Not indicated.	Reported Instruments: Anxiety and depression were measured by the HADS, PCa specific anxiety was measured by the MAX-PC, state trait anxiety was STAT, illness perception measured by the IPQ-R and functional assessment of PCa measured by the FACT-P.	Key Findings: Low levels of anxiety and high HRQOL among AS patients. Patients receiving AS had a fear of recurrence.	Strengths: Use of multiple questionnaires for anxiety and psychological distress. Limitations: All patients from a single practice instead of multiple sites.
Ruane-McAteer et al., (2016)/ Northern Ireland [48]	Purpose: (1) Examine anxiety in non-cancer men, men who received AS for PCa, and men who received active treatment for PCa. (2) Explore patient′s experience of being treated with AS for PCa. Setting: Northern Ireland.	Design: Mixed-methods, Phase 1 quantitative-questionnaires distributed at the Northern Ireland Cancer Centre (NICC) and Belfast City Hospital and follow-up questionnaires every 3 months for 12 months among all groups. Phase 2 qualitative semi structured interviews. Statistics: Descriptives, hierarchical linear modeling, univariate analysis (*t*-test) Sample: 180 each group consisted of 90 men. Response rate: Not listed.	Race/Ethnicity: Not indicated.	Reported Instruments: Demographic data measured by EPQ, depression and anxiety measured with the CES-D, STAI-6, and the MAX-PC, uncertainty measured by the MUIS-C, DCS, DRS, and PCa specific functions were measured by the EPIC.	Key Findings: The study has yet to be implemented and is only a description of what is to occur.	Strengths: The study is novel in that is the first to incorporate baseline data prior to treatments being decided, incorporation of a control group which includes men without cancer in a mixed methods study. Limitations: There are yet to be limitations determined as the study has yet to be implemented.
van den Bergh et al., (2009)/ Rotterdam, the Netherlands and Amsterdam, the Netherlands [43]	Purpose: To examine the levels of decisional conflict, depression, and generic PCa specific anxiety for selecting for AS. Setting: Rotterdam, the Netherlands and Amsterdam, the Netherlands.	Design: Quantitative. Methods: Quantitative, non-RCT. Questionnaires were mailed to the subjects′ home address between May 2007 and May 2008. Statistics: Descriptives, Univariate linear regression analyses, Multivariate linear regression analyses. Sample: 150 eligible, 129 questionnaires returned. Response rate: 86%.	Race/Ethnicity: Not indicated.	Reported Instruments: Depression was assessed with the CES-D. Anxiety was assessed with the abridged STAI-6. PCa-specific anxiety was assessed MAX-PC. General health was assessed using the SF-12.	Key Findings: More than 3/4 of the participants had better scores for the reference values for clinically significant uncertainty based on their treatment decision, depression, generic anxiety, and PCa-specific anxiety. The majority of men had better distress and anxiety scores compared to reference values and other treatments for PCa.	Strengths: High response rate and the use of various questionnaires to assess psychological and mental health issues. Limitations: Patients may have already had low anxiety and distress due to already being on AS. No control group for comparison.
van den Berg et al., (2010)/ The Netherlands [44]	Purpose: To assess anxiety and depression among men receiving AS and their reasons for discontinuation. Setting: Erasmus University Medical Centre.	Design: Quantitative, non-RCT. Methods: Questionnaires were mailed to patients from the PRIAS study with a PCa diagnosis of less than 6 months. Follow-up 9 months after diagnosis. Statistics: Descriptives, multivariate linear regression analysis, paired samples *t*-tests Sample: 150 men at baseline and final of 129. Response rate: 86% for 129 out of 150 and 90% for 108 out of 120.	Race/Ethnicity: Not indicated.	Reported Instruments: Decisional conflict were assessed with the DCS depression was assessed with the CESDS, generic anxiety with the STAI, PCa specific anxiety with the MAX-PC.	Key Findings: Anxiety and distress appeared to be low during the first 9 months of AS. A total of 9 men discontinued AS.	Strengths: High response rate and use of multiple instruments to assess anxiety and depression. Limitations: Patients may have already had decreased rates of anxiety and depression due to already being on AS.
Venderbos et al., (2015)/ Rotterdam, the Netherlands and Amsterdam, the Netherlands [47]	Purpose: To analyze the development of anxiety and distress among men receiving AS. Setting: Rotterdam, the Netherlands and Amsterdam, the Netherlands.	Design: Quantitative, non-RCT. Methods: Questionnaires were mailed to the subjects and follow-up questionnaires were provided at 9 and 18 months. Statistics: Descriptives, Cronbach′s alpha, paired samples *t*-test, and a linear mixed model. Sample: 150 men invited and 129 participated. Response rate: Baseline 86%, 9 month 90%, and 18 month 96%.	Race/Ethnicity: Not indicated.	Reported Instruments: Distress was measured with the DCS, CES-D. General anxiety was measured through the STAI-6 and the MAX-PC measured PCa-specific anxiety. General physical health was assessed with SF-12.	Key Findings: Decreased anxiety and general anxiety and fear of disease progression, among low-risk PCa survivors receiving AS.	Strengths: Use of multiple measures for assessing anxiety and distress. Limitations: Baseline anxiety and distress scores not available, small sample size, and could not compare across time points due to the lack of baseline data.
Watts et al., (2015)/ South, Central and Western England [42]	Purpose: Assess the presence of anxiety and depression among men on AS. Setting: Secondary care prostate cancer (PCa) clinics across South, Central and Western England.	Design: Quantitative, Cross-sectional questionnaire survey. Methods: Participants from 7 sites were mailed the questionnaire. Statistics: Descriptives and logistic regression. Sample: 426 were invited and 313 participated. Response rate: 73.47%.	Race/Ethnicity: White: 302 Afro-Caribbean: 4 Asian: 3 Unknown: 3 Other: 1.	Reported instruments: Depression and anxiety was assessed by the HADS.	Key Findings: Higher rates of anxiety and depression among patients receiving AS than in the general population.	Strengths: Large multi-center examination of anxiety and depression among patients receiving AS. Limitations: Unable to establish causality of anxiety and depression in this population due to statistical methods.

AS = Active surveillance; CES-D = Center for Epidemiologic Studies Depression scale; DCS = Decisional Conflict Scale; DRS = Decisional regret scale; DT = Distress Thermometer; EORTCQLQ-C30 = European Organization for Research and Treatment of Cancer Quality of Life Questionnaire Core 30; EPIC = Expanded Prostate Cancer Index; EQ-5D = General quality of life; EPQ = Eysenck Personality Questionnaire; FACT-P = Functional Assessment of Cancer Therapy – Prostate Version; GAD-7 = General Anxiety Disorder scale 7; HRQoL = Health related quality of life; IPQ-R = illness perception questionnaire-revised; Mini-MAC = Mini Mental Adjustment to Cancer; PCa =Prostate cancer; MAX-PC = Memorial Anxiety Scale for Prostate Cancer; MUIS-C = Mishcel Uncertainty form; PHQ-9 = Patient Health Questionnaire; PRIAS = Prostate Cancer Research International Active Surveillance; QoL = Quality of Life; QLI = Quality of Life Index; RP = Radical prostatectomy; SF-12 = Medical Outcomes Study 12-item short-form health survey; SCL-90 = Symptom Checklist 90; STAI = State-Trait Anxiety Inventory for Adults; UCLA-PCI = UCLA Prostate Cancer Index.
